# Introduction to the Proceedings of the Sixth Annual Conference on Native American Nutrition

**DOI:** 10.1016/j.cdnut.2026.109404

**Published:** 2026-09-22

**Authors:** Mindy S Kurzer

**Affiliations:** Department of Food Science and Nutrition, University of Minnesota, Saint Paul, MN, United States

The Sixth Annual Conference on Native American Nutrition: Resurgence of Indigenous Foodways, was held at Mystic Lake Casino Hotel in Prior Lake, MN, 10 to 12 September, 2023. It was cohosted by the University of Minnesota Healthy Foods, Healthy Lives Institute, and the Oklahoma State University Center for Indigenous Health Research and Policy. Major sponsors included the Minnesota Department of Human Service and the Tamalpais Trust. Additional significant sponsors included Feeding America, Indigenous Food and Agriculture Initiative, Johns Hopkins University Bloomberg American Health Initiative, Native American Food Sovereignty Alliance, Newman’s Own Foundation, North American Traditional Indigenous Food Systems, and the University of Hawai’i Center for Indigenous Innovation and Health Equity.

This conference was the sixth in a series of conferences that have focused on using the strengths of Indigenous communities to improve health through food. A major theme of the conference was supporting Indigenous food systems by improving knowledge of the healthfulness of traditional foods and how to grow, distribute, and prepare them. Underlying this is the understanding that for Indigenous communities, food-related health is a broad concept that goes well beyond a biomedical understanding of nutrition. While a biomedical approach is narrow and generally centered on individuals, an Indigenous approach is holistic and systemic, centered on the family and community, and intertwined with many other factors such as identity, language, relationship with land, culture, ancestral knowledge, decolonization, and planetary health.

The website of the conference contains the complete program with speaker bios and abstracts, a complete list of all sponsors, testimonials, and a link to videos of all presentations [1]. Sessions included “Indigenous Perspectives on the Role of Food in Health and Wellbeing,” “Model Tribal Programs,” “Nurturing Youth and Junior Professionals,” “The Power of Indigenous Knowledge to Move Science Forward,” and “Food and Nutrition Policy in Action.” In addition to the invited speakers and panelists, there was a session of lightning talks (Wakinyan Tunyanpi Woglaka) selected from abstracts submitted by attendees. Topics of these brief talks included Indigenous diabetes prevention models, cooking preparation classes adapted for Indigenous communities, novel Supplemental Nutrition Assistance Program-Education (SNAP-Ed) programs geared toward Indigenous communities, incorporation of traditional foods into school meals, healthy infant and family nutrition, usage of community-based participatory research to inform policy, connection of Native American growers to food pantries accessed by Native communities, adaptation of produce prescription programs for Indigenous communities, and food systems approaches to food security and Indigenous food sovereignty.

The sixth conference was a great success. There were 752 attendees from 38 states plus Washington, DC, and 5 Canadian provinces. Most attended in-person, although for the first time, there was a virtual option. Approximately 45% of all conference participants claimed an affiliation to a Native tribe or community, and >100 tribes were represented. Although all attendees worked or were particularly interested in issues of food-related health of Indigenous Peoples, attendees represented a broad range of affiliations. One-quarter of the attendees were tribal officials, employees, or community members; one-quarter were nutrition educators, public health workers, or clinicians; one-quarter were researchers, students, higher education employees, or federal state or local government employees; and the remaining quarter were made up of nonprofit employees, consultants or business owners, and funders.

These proceedings highlight a few of the presentations at the sixth conference. The 6 papers discuss novel definitions of nutrition through the lens of Indigenous worldviews [2], model SNAP-Ed programs for Indigenous communities [3], development of a novel diabetes prevention program for Indigenous women [4], adaptation of a SNAP-Ed cooking and nutrition education program for Indigenous communities [5], unique food-related challenges and solutions of urban Indigenous communities [6], and the health benefits of traditional foods [7].

The guest editor thanks the co-hosts and sponsors for their generous support of this conference series, as well as the planning committee members (listed on the conference website), who worked tirelessly to create excellent programming. The proceedings of the seventh annual conference, held in 2025 [8], will be published in 2027. The eighth conference in this series is being planned to take place in the fall of 2027. We look forward to continuing hosting this conference series to highlight the strengths of Indigenous communities and their food systems for the continued health of future generations.

## Author contributions

The sole author was responsible for all aspects of this manuscript.

## Declaration of Generative AI and AI-assisted technologies in the writing process

AI was not used in the writing process for this manuscript.

## Funding

These proceedings were supported by the University of Minnesota’s Healthy Foods, Healthy Lives Institute, and the Oklahoma State University Center for Indigenous Health Research and Policy.

## Conflict of interest

MSK is an Academic Editor for *Current Developments in Nutrition* and played no role in the Journal’s evaluation of this manuscript.
